# Transcriptomic Profiling Reveals the Involvement of the Phenylpropanoid–Lignin Pathway in the Response of Maize Roots to Zinc Stress

**DOI:** 10.3390/plants14111657

**Published:** 2025-05-29

**Authors:** Ying Zhou, Tianyu Gu, Yan Gao, Jingtao Qu, Hongjian Zheng, Yuan Guan, Jiashi Peng

**Affiliations:** 1 School of Life and Health Sciences, Hunan University of Science and Technology, Xiangtan 411201, China; zhouying202505@163.com (Y.Z.); guty@hnust.edu.cn (T.G.); blessedgy@hnust.edu.cn (Y.G.); 2 CIMMYT-China Specialty Maize Research Center, Crop Breeding and Cultivation Research Institute, Shanghai Academy of Agricultural Sciences, Shanghai 201403, China; qujingtao@saas.sh.cn (J.Q.); hjzh6188@163.com (H.Z.); 3 Hunan Key Laboratory of Economic Crops Genetic Improvement and Integrated Utilization, Hunan University of Science and Technology, Xiangtan 411201, China; 4 Hunan Province University Key Laboratory of Ecological Remediation and Safe Utilization of Heavy Metal-Polluted Soils, Hunan University of Science and Technology, Xiangtan 411201, China

**Keywords:** zinc detoxification, *Zea mays*, phenylpropanoid, lignin, peroxidase, cell wall, transcriptome

## Abstract

Zinc (Zn) is an essential micronutrient required for plants to perform various metabolic functions, and plant responses to Zn deficiency have been extensively studied. However, excessive levels of Zn in soil can induce toxic effects in plants, posing a substantial challenge to global agricultural productivity. Consequently, elucidating the response mechanisms of crop plants to excessive Zn toxicity is currently of great significance. In this study, seedlings of maize inbred line B73 were exposed to excessive Zn treatment, and transcriptomic profiling of the roots was conducted at 0, 2, 6, 12, 24, and 48 h post-treatment. In addition to changes in the expression of genes encoding zinc-regulated, iron-regulated transporter-like protein (ZIP), metal tolerance protein (MTP), and yellow stripe-like (YSL) transporter family members involved in Zn transport, we observed that differentially expressed genes (DEGs) were significantly enriched in the phenylpropanoid–lignin metabolic pathway across all treatment stages, including the early (2 and 6 h), middle (12 and 24 h), and late (48 h) stages of Zn treatment. Among the 11 core structural enzyme-encoding genes involved in monolignols biosynthesis from phenylalanine in this pathway, the expression of eight of them was altered by Zn treatment. Additionally, genes encoding peroxidase (POD), which are responsible for the polymerization of monolignols into lignin, demonstrated extensive changes across all treatment stages, particularly at the late stage. The expression levels of these key enzyme genes were further validated using quantitative real-time PCR. Correspondingly, the activity of POD enzymes and the lignin content both significantly increased in Zn treated roots. These findings suggest that the phenylpropanoid–lignin metabolic pathway plays a crucial role in maize root responses to excessive Zn stress.

## 1. Introduction

Zinc (Zn) is an essential micronutrient for plants, functioning primarily as a cofactor for various enzymes and stabilizing proteins such as Zn-finger proteins [1]. However, the excessive accumulation of Zn in soil, caused by the extensive use of fertilizers and industrial pollution, is currently recognized as a global issue [2,3]. When Zn accumulates excessively in soil, it inhibits the absorption of other nutrients such as Ca, P, K, and Fe by plant roots and causes excessive Zn accumulation in plants [4]. The toxicity threshold for Zn in plants ranges between 100 and 300 μg/g dry weight, depending on the species and physiological state [5]. Excessive Zn accumulation in plants leads to the production of reactive oxygen species (ROS), causing lipid peroxidation and growth inhibition [6]. As a heavy metal, Zn toxicity in plants is primarily mitigated through chelation or vacuolar compartmentalization. Chelators such as nicotianamine (NA), phytochelatins, and metallothioneins, as well as transporters from the family of cation diffusion facilitators (CDFs) and zinc-regulated, iron-regulated transporter-like proteins (ZIPs) that sequestrate Zn into vacuoles, play significant roles in these processes [5,7,8,9,10]. Moreover, the removal of ROS mediated by glutathione is also important for alleviating the stress caused by excessive Zn toxicity [11].

The phenylpropanoid metabolic pathway is one of the most important secondary metabolic pathways in plants. The protective secondary metabolites yielded in this pathway, such as flavonoids and lignin, are important for environmental adaptation. Eleven core structural enzymes involved in monolignols biosynthesis from phenylalanine, the initial substrate of phenylpropanoid metabolism, have been identified. These include L-phenylalanine ammonia-lyase (PAL), 4-hydroxycinnamate CoA ligase (4CL), cinnamate 4-hydroxylase (C4H), cinnamoyl CoA reductase (CCR), cinnamyl alcohol dehydrogenase (CAD), coumarate 3-hydroxylase (C3H)/coumaroyl shikimate 3′-hydroxylase (C3′H), ferulate/coniferaldehyde 5-hydroxylase (F5H), caffeate/5-hydroxy-coniferaldehyde 3/5-O-methyltransferase (COMT), caffeoyl CoA 3-O-methyltransferase (CCoAOMT), hydroxycinnamoyl CoA: shikimate hydroxycinnamoyl transferase (HCT), and caffeoyl shikimate esterase (CSE) [12]. Subsequently, monolignols are polymerized to form lignin under the catalysis of peroxidase (POD) [13]. Lignin is one of the main components of the plant cell wall and is closely associated with heavy metal detoxification. Numerous functional groups in lignin, such as hydroxyl, carboxyl, and methoxyl, can bind heavy metal ions and reduce their entry into the cytoplasm [13,14]. To date, there have been few reports on Zn stress resistance mediated by the phenylpropanoid–lignin pathway. Studies on watermelon have demonstrated that BR treatment significantly induces the expression of key genes in the phenylpropanoid–lignin metabolic pathway, such as PAL, 4CL, CCR, and CCoAOMT, increasing lignin content by 29.26% and thereby enhancing tolerance to Zn stress in watermelon [15].

Maize (*Zea mays*) is a globally important food and feed crop, and the biofortification of Zn in maize has been a concern in the scientific community. However, the regulation of Zn accumulation in maize remains largely unknown. The expression of *Natural Resistance Associated Macrophage Protein1* (*ZmNRAMP1*) and members of maize *Zrt*, *Irt-like Protein* (*ZmZIP*), including *Iron-regulated transporters1* (*ZmIRT1*), *ZmZIP4*, *ZmZIP5*, *ZmZIP7*, *ZmZIP8*, *ZIP-like protein1* (*ZmZLP1*) is sensitivity to Zn treatment, and these genes are thought to play a role in the regulation of Zn homeostasis in maize [16,17,18]. Recent studies have shown that overexpression of *ZmLazarus1-4* (*ZmLAZ1-4*) and yellow *stripe-like transporter2* (*ZmYSL2*), which belong to the Domain of Unknown Function (DUF) 300 family and YSL family, respectively, can also increase Zn accumulation in maize [19,20]. However, the research on how maize responds to excessive Zn stress remains limited. This study analyzed the transcriptomic response of maize roots at different times following excessive Zn treatment and found that differentially expressed genes (DEGs) were enriched in the phenylpropanoid–lignin metabolic pathway. Furthermore, the detection of key enzyme gene expression in the pathway and lignin content in the roots following Zn treatment demonstrated the significant role of the phenylpropanoid–lignin metabolic pathway in the root’s response to Zn stress. These findings provide new insights into the mechanism of adaptation to excessive Zn stress in maize.

## 2. Results

### 2.1. Zn Treatments and Transcriptome Sequencing

**Figure 1:**
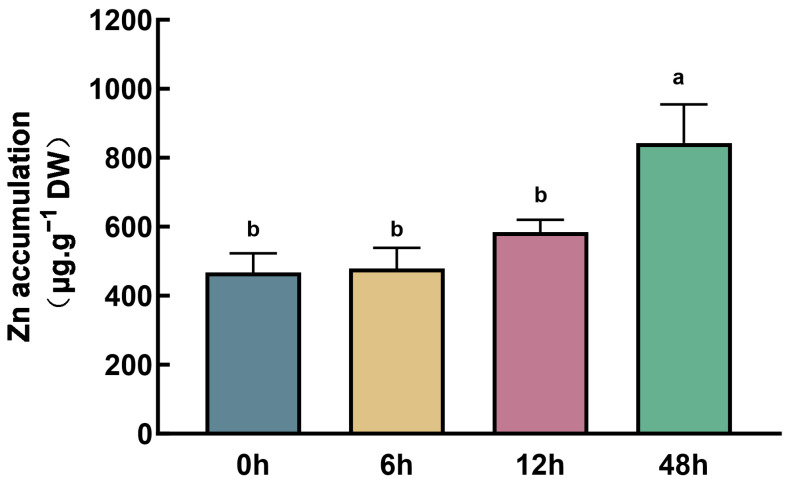
Zn content in maize roots after Zn treatment at different times. Different lowercase letters indicate significant differences among treatments (*p* < 0.05).

Maize seedlings with uniform growth, cultured hydroponically, were divided into six groups, and Zn was added to each group at different time points to reach a concentration of 150 μM. The samples were subjected to treatment for 0, 2, 6, 12, 24, and 48 h, respectively. To ensure consistency and avoid variations caused by circadian rhythms and plant age, each sample was treated with Zn at a distinct time, but all samples were harvested at the same time. No visible Zn stress phenotype was observed in any of the treated plants. However, the Zn content in the roots increased with the extension of the treatment time. After 48 h of treatment, the Zn concentration in the roots was approximately twice that of the untreated maize (Figure 1).

To identify the pathways critical for the response to Zn stress in maize, transcriptomic analysis was conducted on all root samples, with three biological replicates per group. Each sample replicate generated over 40 million clean reads, and the Q30 value for each sample exceeded 94.1%. More than 94.9% of the reads from each sample were successfully mapped to the reference genome of maize (Supplementary Table S1). Further correlation analysis among sample replicates suggested strong consistency across the three replicates of the samples (Supplementary Figure S1). The average expression abundance of genes in all samples was comparable, while the variation in gene expression differed, suggesting that Zn treatments at different times caused transcriptional changes in the roots of maize (Supplementary Figure S2). Together, these data demonstrate the high randomness and reliability of the sequencing data.

### 2.2. Identification of Differentially Expressed Genes (DEGs) at Different Treatment Stages

**Figure 2:**
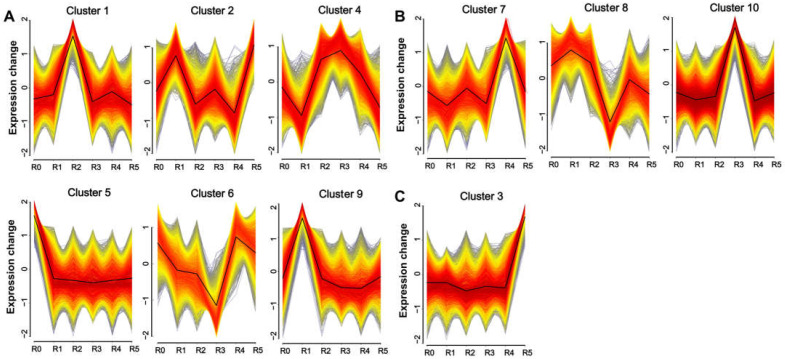
Distinct patterns of gene expression changes under zinc treatment at different times. (**A**) Genes with significant changes during the early-response stage (ERS): 2 h (R1), 6 h (R2). (**B**) Genes with significant changes during the middle-response stage (MRS): 12 h (R2), 24 h (R4). (**C**) Genes with significant changes during the late-response stage (LRS): 48 h (R5).

To investigate the temporal response of the maize root transcriptome to Zn stress, we performed cluster analysis using Mfuzz, resulting in the identification of 10 distinct clusters (Figure 2). Our primary focus was to identify the pathways responsive to Zn treatment at different time periods in maize. Therefore, according to the time point when the DEGs in the cluster began to change, they were classified into three patterns: early-response stage (ERS), middle-response stage (MRS), and late-response stage (LRS). The ERS pattern encompassed Clusters 1, 2, 4, 5, 6, and 9, in which gene expression levels were significantly altered within 2 or 6 h of zinc exposure. The MRS pattern included Clusters 7, 8, and 10, with significant changes observed after 12 or 24 h of zinc exposure. The LRS pattern comprised Cluster 3, where gene expression levels were markedly altered after 48 h of zinc treatment.

**Figure 3:**
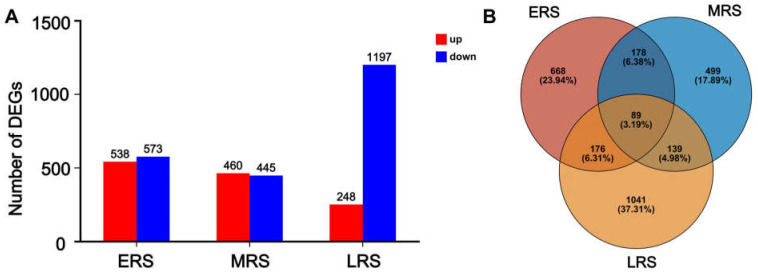
The number of DEGs in different treatment stages. (**A**) The number of upregulated and downregulated genes in the early stage, middle stage, and late stage. (**B**) Venn diagram showing the overlapping and unique DEGs among the early stage, middle stage, and late stage.

Using untreated maize (0 h treatment) as the control, a total of 538 genes were upregulated and 573 genes were downregulated during the ERS, while 460 genes were upregulated and 445 genes were downregulated during the MRS. After 48 h of Zn treatment, 248 genes were upregulated and 1197 genes were downregulated (Figure 3A). Furthermore, 668, 499, and 1041 genes exhibited specific differential expression in the ERS, MRS, and LRS, respectively. A total of 89 genes demonstrated altered expression across all treatment stages (Figure 3B).

### 2.3. Zn Stress-Induced Extensive Changes in Phenylpropanoid–Lignin Pathway in Roots

KEGG analysis of all DEGs indicated that “phenylpropanoid biosynthesis” was the most significantly enriched pathway (Supplementary Table S2). Phenylpropanoid metabolism produces end products such as flavonoids, hydroxycinnamic acid esters, and the precursors of lignin and tannins, which play a crucial role in plant resistance to adverse environmental stresses [21]. In the GO enrichment results, DEGs were also significantly enriched in the “phenylpropanoid metabolic process” and “phenylpropanoid biosynthetic process” (Supplementary Table S3). Moreover, cell wall-related pathways were significantly enriched, especially lignin-related pathways such as the “lignin metabolic process”, “lignin biosynthetic process”, and “lignin catabolic process”. Consistently, the POD-related pathways involved in catalyzing the synthesis of lignin from monolignols, which are products of phenylpropanoid metabolism, were also significantly enriched (Supplementary Table S3). These observations indicate that the phenylpropanoid–lignin pathway is a major pathway involved in the response to excessive Zn stress in maize roots.

**Figure 4:**
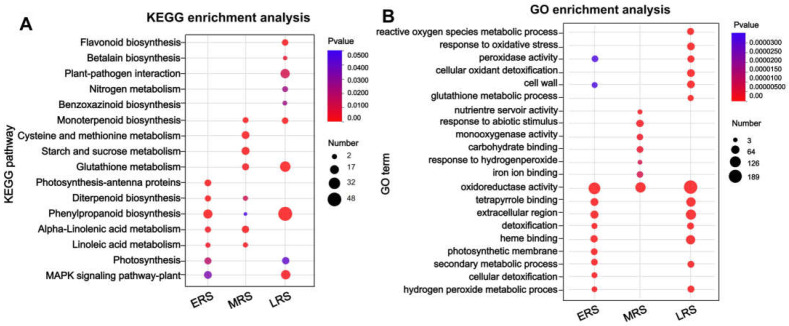
Functional enrichment analysis of DEGs at different stages of Zn treatment in maize roots. (**A**) KEGG enrichment analysis; (**B**) Go enrichment analysis.

Here, we focus on pathways that respond early to Zn treatment and continue to function throughout the treatment period, in which gene expression in these pathways as altered across all three stages of Zn treatment. KEGG analysis of the 89 DEGs that changed during the ERS, MRS, and LRS (Figure 3B) showed that they were mainly enriched in pathways such as “phenylpropanoid metabolism” and amino acid and small peptide metabolism (Supplementary Figure S2). These findings suggest that the phenylpropanoid metabolism pathway continuously responds to Zn stress and is involved in Zn detoxification in maize roots. Subsequently, KEGG and GO enrichment analyses were conducted on the DEGs identified in the ERS, MRS, and LRS, respectively. In the KEGG enrichment analysis, the “phenylpropanoid biosynthesis” pathway was significantly enriched at all three stages of Zn treatment (Figure 4A), while pathways such as “POD activity”, “cell wall”, and “oxidoreductase activity” were also significantly enriched in the GO analysis (Figure 4A). The results further indicate that the phenylpropanoid–lignin pathway is involved in the response to excess Zn stress from the early to late stages of treatment and continues to play a role in maize roots.

**Figure 5:**
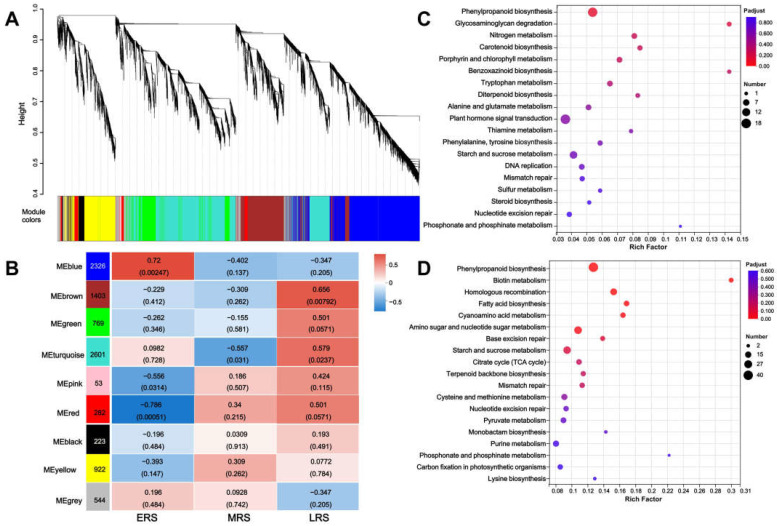
Weighted gene co-expression network analysis (WGCNA) of DEGs in maize roots. (**A**) Cluster analysis of WGCNA modules (each color represents a module). (**B**) Correlation between different time stages based on different modules. (**C**,**D**) KEGG analysis of the blue module (**C**) and the brown module (**D**).

Weighted gene co-expression network analysis (WGCNA) categorizes genes with similar expression patterns into modules and analyzes linkages between gene modules and sample traits [22], which helps to identify gene modules closely associated with different treatment times of Zn. Given this, WGCNA was conducted to classify DEGs into modules, and a total of 9 co-expression gene modules were generated (Figure 5A). We subsequently detected the correlations between co-expression modules and Zn treatment stages. Among all nine modules in Figure 5B, the blue module contained 2326 genes and was most positively correlated with the early stage of Zn treatment. Meanwhile, the brown module contained 1403 genes and was most positively correlated with the late stage of Zn treatment. Therefore, these two representative modules were selected, and functional annotation was performed using KEGG pathway analysis. Interestingly, in the results of KEGG analysis of both modules, the “phenylpropanoid biosynthesis” pathway was the most significantly enriched (Figure 5C,D). These results further suggest that the phenylpropanoid biosynthesis pathway may function continuously throughout the maize root response to Zn treatment.

**Figure 6:**
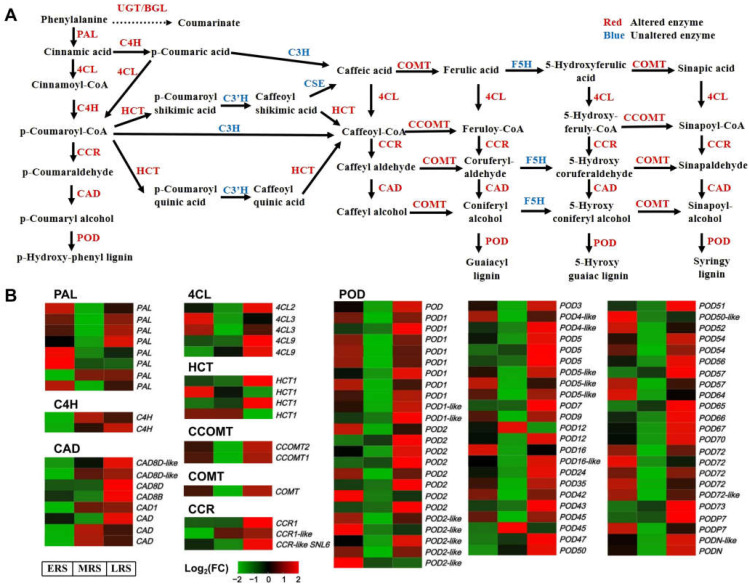
Expression of DEGs in maize roots related to the phenylpropanoid–lignin pathway. (**A**) Metabolic diagram of the phenylpropanoid–lignin pathway. (**B**) Expression changes of DEGs in the phenylpropanoid–lignin pathway at different stages of Zn treatment.

There are 11 core structural enzymes responsible for the synthesis of lignin precursors from phenylalanine in the phenylpropanoid–lignin pathway. The expression levels of genes encoding 8 of these enzymes was altered at different stages of Zn treatment, including PALs (8 transcripts), C4Hs (2 transcripts), CADs (8 transcripts), 4CLs (5 transcripts), HCTs (4 transcripts), CCOMTs (2 transcripts), CCRs (3 transcripts), and COMTs (1 transcripts) (Figure 6). These genes responded during the ERS, MRS, and LRS (Figure 6B), enabling the phenylpropanoid–lignin pathway to continuously function throughout the three stages of Zn treatment. POD is a key enzyme that catalyzes the synthesis of lignin from lignin precursors. Correspondingly, the expression of 70 genes encoding POD also changed at different stages of Zn treatment (Figure 6B). Among these DEGs, genes that were upregulated in the early stage were primarily those located upstream in the phenylpropanoid metabolism pathway, such as *PALs* and *4CLs*, while those upregulated in the middle stage were mainly midstream genes in the pathways, including *C4Hs* and *CADs*. Genes upregulated in the later stage were mainly those located downstream in the pathway, such as *HCTs*, *CCOMTs*, *COMTs*, *CCRs*, and the *POD* genes that mediate lignin synthesis. It is worth noting that flavonoid metabolism, another branch of phenylpropanoid metabolism, also showed significant changes. However, fewer genes were affected overall, and the changes occurred almost exclusively during the LRS (Supplementary Figure S4).

### 2.4. Validation of Expression Changes of Enzyme Genes in the Phenylpropanoid–Lignin Pathway by qRT-PCR Analysis

**Figure 7:**
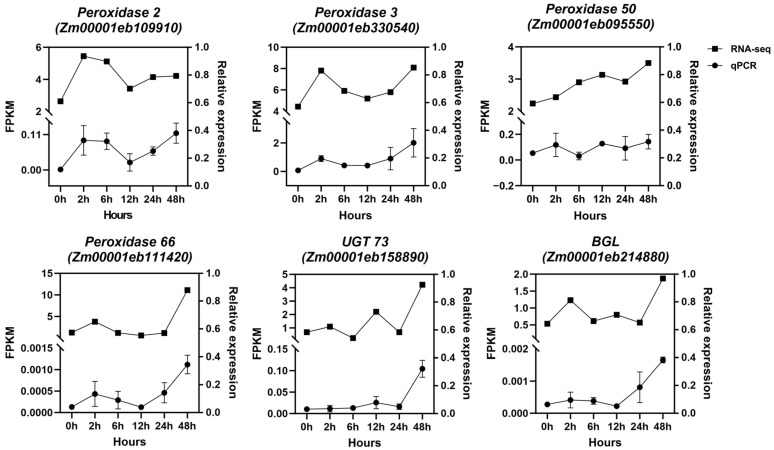
Relative expression analysis of selected DEGs by qRT-PCR. *ZmGAPDH* was used as the internal control, and three independent replicates were adopted for qRT-PCR.

To validate the expression patterns of RNA-seq data, qRT-PCR analysis was performed on 6 randomly selected transcripts from phenylpropanoid–lignin pathway: *ZmPOD2*, *ZmPOD3*, *ZmPOD50*, *ZmPOD66*, *ZmBGL*, and *ZmUGT73*. The relative expression trends of all the genes in response to the Zn treatment at different stages were consistent with the results of RNA-seq data (Figure 7). This strong correlation validates the reliability and accuracy of the RNA-seq data obtained in this study and further confirms the involvement of the phenylpropanoid–lignin pathway in the maize root response to excess Zn stress.

### 2.5. The Activity of POD Enzymes and the Lignin Content Increased with Zn Exposure

**Figure 8:**
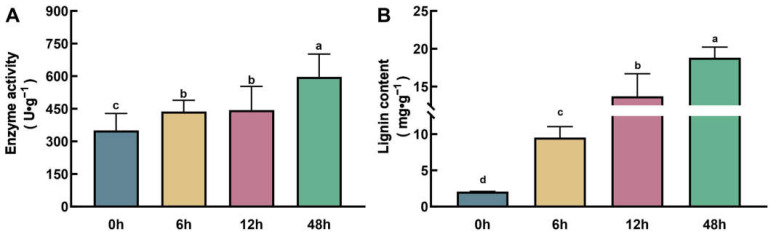
Measurement of PODs activity (**A**) and the lignin content (**B**) in maize roots with Zn exposure. Different lowercase letters indicate significant differences among treatments (*p* < 0.05).

RNA-seq data indicated that transcripts encoding PODs exhibited extensive and significant changes at all stages of Zn treatment (Figure 4B, Supplementary Table S3). PODs are not only involved in maintaining ROS homeostasis functioning in plant stress response but also serve as key enzymes in catalyzing the polymerization of lignin from monolignols, the end products of phenylpropanoid metabolism [21]. Therefore, we first determined the enzyme activity of PODs in the roots of maize at 0 h, 6 h, 12 h, and 48 h after Zn treatment. The results showed that with increasing Zn treatment duration, the enzyme activity of PODs was significantly enhanced. After 48 h of treatment, the enzyme activity of PODs increased by 34% compared with the untreated control (Figure 8A). In addition, we also determined the content of lignin in the roots. Correspondingly, the content of lignin also significantly increased with the extension of Zn treatment time. Compared with the untreated control, the content of lignin in the roots increased 3.6-fold, 5.6-fold, and 18.8-fold after 6 h, 12 h, and 48 h of treatment, respectively (Figure 8B). These results further confirm that the phenylpropanoid–lignin pathway is involved in the response to excessive Zn stress and the process of Zn detoxification in maize roots.

## 3. Discussion

Zn is an essential element for both plants and humans. As a major global food crop, maize is an important source of Zn for human populations. Research on the transcriptome profiles of maize in response to Zn has primarily focused on the response to Zn deficiency. Under Zn deficiency, the expression of ZIP transporter genes and *NA synthase* (*NAS*) genes involved in Zn absorption and transport is upregulated, while the expression of genes related to ROS, cell walls, and DNA methylation is downregulated [23,24]. In addition, genes related to chlorophyll synthesis and hormone pathways also respond to Zn deficiency [18,25]. Although Zn toxicity in plants is less widespread than Zn deficiency, nevertheless, excessive Zn in the soil has now become an important limiting factor for global agriculture [4,26]. Noxious levels of Zn can lead to a decline in soil fertility and hinder plant growth and development, including reduced photosynthetic and respiratory rates, imbalanced mineral nutrition, and enhanced generation of ROS. Therefore, studying the transcriptomic response of maize to excessive Zn will shed light on understanding the mechanism of Zn tolerance and support breeding efforts aimed at enhancing tolerance to Zn contamination.

In this study, we analyzed the transcriptome of maize roots treated with 150 μM Zn for varying durations. We did not use higher concentrations of Zn to treat maize seedlings, as they would cause obvious stress responses in maize seedlings [27], potentially increasing the occurrence of secondary responses in the transcriptome detection. Moreover, we believe that the relatively mild Zn stress applied in this study more closely reflects actual conditions. The expression alteration of genes encoding potential Zn transporters and chelators, which functioned essentially in Zn uptake, transport, and chelation processes, were analyzed. We found that the expression of genes encoding the transporters from the family of ZIP (8 transcripts), HMA (heavy metal ATPase) (3 transcripts), MTP (metal tolerance protein) (1 transcripts), VIT (vacuolar iron transporter) (2 transcripts), YSL (yellow stripe-like) (6 transcripts), ZIF (zinc-induced facilitator) (1 transcripts), as well as the genes encoding the chelators from the family of NAS (2 transcripts) and MT (metallothionein) (4 transcripts), were extensively altered by Zn treatment. These observations not only suggest that these Zn transporters and chelators may be involved in the resistance to zinc stress in maize, but they also indicate that our zinc treatment conditions caused extensive responses in maize roots.

Here, we focused more on the pathways that can continuously respond and function in maize roots throughout the process of Zn treatment. Therefore, we classified DEGs into the early, middle, and late stages of Zn treatment according to the time point when they began to change, but we did not distinguish whether their expression was upregulated or downregulated (Figure 2). In the results of the transcriptome analysis, multiple sets of data all pointed to the phenylpropanoid–lignin pathway playing a continuous role in the response to Zn treatment in maize roots. Firstly, both the KEGG and GO enrichment analyses of all DEGs showed that these DEGs were significantly enriched in “phenylpropanoid metabolism” and cell wall-related pathways (especially the lignin pathways) (Supplementary Tables S2 and S3). Secondly, to identify the pathways that responded early and continuously functioned in maize under Zn treatment, we divided the DEGs that altered from 0 to 48 h of Zn treatment into three stages: ERS, MRS, and LRS (Figure 2). The 89 DEGs common to these three stages were also significantly enriched in “phenylpropanoid metabolism” (Supplementary Figure S3). Thirdly, the KEGG and GO enrichment analysis results of the DEGs in the ERS, MRS, and LRS periods also showed that “phenylpropanoid biosynthesis”, “POD activity”, and cell wall-related pathways were significantly enriched (Figure 4). Fourthly, in the WGCNA analysis, the DEGs in the gene modules most associated with the early and late stages of Zn treatment were significantly enriched in the “phenylpropanoid biosynthesis” in the KEGG analysis (Figure 5). In addition, by analyzing the 11 core structural enzymes for synthesis of lignin precursors from phenylalanine in the phenylpropanoid metabolism pathway, we found that the expression level of the genes encoding eight of these enzymes was altered at different stages of Zn treatment (Figure 6). The expression of the key enzyme gene PODs that catalyze the polymerization of lignin precursors into lignin also changed in response to different stages of Zn treatment (Figure 6). Consistently, the expression of member genes in the phenylpropanoid–lignin pathway changed across different time points under Zn treatment. Upstream genes in the pathway were first upregulated in ERS, followed by the downstream genes being upregulated in MRS and LRS in sequence (Figure 6). These results of transcriptome analysis strongly suggest that the phenylpropanoid–lignin pathway responds early to excessive Zn stress in maize and functions continuously during Zn detoxification. Subsequent measurement of POD enzyme activity and lignin content also found that they increased with the extension of Zn treatment time, further verifying that the phenylpropanoid–lignin pathway continuously plays a role in the response to Zn treatment in maize roots.

The cell wall is an important structure that helps plants resist heavy metal toxicity. Lignin is one of the main components of the secondary cell wall in plants. It is polymerized from monolignols, the end products of phenylpropanoid metabolism. Therefore, the phenylpropanoid–lignin pathway is widely involved in the process of plants resisting heavy metal stress [13]. Heavy metal stress can induce the expression of key genes in the lignin synthesis pathway, increasing the lignin content in the secondary cell wall, thereby increasing the thickness of the cell wall [28,29,30,31,32]. Meanwhile, because lignin polymers contain a large number of functional groups (hydroxyl, carboxyl, methoxy, etc.), they can bind to various heavy metal ions, such as Cu^2+^, Cd^2+^, Pb^2+^, Zn^2+^, etc., thereby reducing the uptake by cells and mitigating the toxicity of heavy metals [33]. It has been reported that lignin is involved in Zn homeostasis. Guo et al. reported that lignin can bind to Zn^2+^, and carboxyl and phenolic groups are the primary binding sites [33]. In the Zn hyperaccumulator *Thlaspi caerulescens*, the expression abundance of genes related to lignin synthesis is significantly higher than that of its close relative Arabidopsis, and can be induced by excessive Zn treatment, resulting in significant differences in lignification between the roots of *T. caerulescens* and Arabidopsis [31]. In this study, Zn stress significantly induced the phenylpropanoid–lignin pathway in the roots of maize, resulting in a 3.6-fold, 5.6-fold, and 18.8-fold increase in lignin content in the roots after 6 h, 12 h, and 48 h of Zn treatment, respectively (Figure 8B). We speculate that the increase in lignin synthesis enhances the chelation and retardation of Zn by the cell wall, reducing the transport of Zn into the root cells and to the shoots, thereby playing a role in resisting excessive Zn stress.

## 4. Materials and Methods

### 4.1. Plant Materials and Zn Treatment

The material used in this study was maize inbred line B73. After germinating, the seeds were placed on germination paper and cultured until reaching the two-leaf, one-heart stage. Then, they were transferred to half-strength Hoagland solution (½HS) for 7 days, during which the nutrient solution was replaced every 2–3 days. Seedlings with consistent growth were transferred to ½ HS or ½ HS supplemented with ZnSO_4_ to 150 μM. Plants were harvested at 0, 2, 6, 12, 24, or 48 h after treatment, and roots were isolated for metal content measurements and RNA-seq analysis.

### 4.2. RNA Extraction, Library Construction, and RNA Sequencing

Root samples from each treatment group were ground in liquid nitrogen, and total RNA was extracted using an RNA extraction kit (DP441, Tiangen Biotech, Beijing, China). The RNA was assessed by electrophoresis, and RNA quality metrics (RQN) were measured using an Agilent Bioanalyser (Fragment Analyzer 5300, M5311AA, Agilent Technologies, Santa Clara, CA, USA). The library was constructed using the Illumina^®^ Stranded mRNA Prep, Ligation (Illumina, San Diego, CA, USA) method. All the RNA samples met library construction requirements of total RNA ≥ 1 μg, concentration ≥ 30 ng/μL, RQN > 6.5, and OD260/280 between 1.8 and 2.2. Briefly, the qualified RNA underwent Oligo dT enrichment for mRNA, followed by fragmentation of mRNA, reverse transcription to cDNA, and adapter ligation. Finally, fragment selection and library enrichment were performed, which involved purifying the adapter-ligated products and fragment sorting [34]. The sorted products were then subjected to PCR amplification, and the final library was purified and sequenced on the NovaSeq X Plus platform.

### 4.3. Reads Mapping and Quantification of Gene Expression Levels

Sequencing data quality control was conducted using fastp (https://github.com/OpenGene/fastp (accessed on 18 November 2022)) to remove low-quality reads (clean reads criteria: Phred quality score ≥ 20, length ≥ 50 bp). The cleaned data after quality control were aligned with the maize reference genome (reference genome version: Zm-B73-REFERENCE-NAM-5.0, reference genome source: http://plants.ensembl.org/Zea_mays/Info/Index (accessed on 18 November 2022)) using HiSat2 software. The expression level of each transcript was calculated according to the fragments per kilobase of exon per million mapped fragments (FPKM) method. RNA-Seq by Expectation-Maximization (RSEM) was used to quantify gene abundances [35].

### 4.4. Identification of Differentially Expressed Genes

Gene and transcript expression levels were quantitatively analyzed using the RSEM (Version 1.3.3) software. After obtaining the gene read counts, DESeq2 software (Version 1.24.0) was used for differential expression analysis of differentially expressed genes (DEGs) [36]. FDR < 0.05 and |log2FC| ≥ 1.

### 4.5. Mfuzz Analysis

Mfuzz (Version 2.6.0) is software designed for studying gene expression time trends in transcriptomic data with time-series characteristics [37]. Its core algorithm is a soft clustering algorithm based on Fuzzy C-Means, which assigns the same gene to different clusters, using the Membership value to differentiate the core expression patterns of that gene and the representative degree it shows in different clusters.

### 4.6. Functional Enrichment Analysis

Gene Ontology (GO) enrichment analysis: Differentially expressed genes were functionally annotated using GOATOOLS (Version 0.6.5), with a significance level set to FDR (False Discovery Rate) < 0.05. Kyoto Encylopedia of Genes and Genomes (KEGG) pathway enrichment analysis was performed using the Python scipy package (Version 1.0.0).

### 4.7. Weighted Gene Co-Expression Network Analysis (WGCNA)

WGCNA was performed using the R WGCNA package (Version 1.63). A total of 9123 genes were obtained from 18 samples after screening out the genes with a mean FPKM value and variable coefficient less than 1 and 0.1, respectively. Modules were identified by setting the following parameters: a soft power β of 9, a minimum model size of 30, and a merge cut height of 0.25. Gene significance (GS) values were calculated using Pearson’s correlation to evaluate the correlation between genes and treatment stages. Module membership (MM) was used to assess the association between the expression profiles of genes and corresponding modules.

### 4.8. Determination of Metal Content

The maize plant samples were washed with deionized water and an EDTA solution as described previously [38,39,40]. Roots were isolated, dried at 60 °C until they reached a constant weight, and then ground into a powder. Approximately 2–5 mg of each sample was weighed and transferred to a digestion tube. Next, 1 mL of 70% nitric acid was added to the digestion tube, which was then placed in a TOPEX intelligent microwave digestion instrument (PreeKem, Shanghai, China) for digestion. Finally, the solution was diluted to a volume of 10 mL with deionized water [41]. The prepared solution was analyzed for metal content using an inductively coupled plasma mass spectrometer (NexION 2000, Perkin Elmer, Waltham, MA, USA).

### 4.9. Determination of Antioxidant Enzyme Activity and Lignin Content

Peroxidase (POD) activity was determined using the guaiacol method [42], and lignin content was measured using the UV absorption method [43].

### 4.10. Quantitative RT-PCR (qRT-PCR)

The qRT-PCR was performed as described previously [44]. The total RNA used was from the same batch as the RNA-seq analysis. Primers were designed using Primer Premier 5 software, and their sequences are listed in Supplementary Table S4. Gene expression levels were measured using an ABI7500 real-time fluorescent quantitative PCR (ABI7500, Thermo Fisher Scientific, Waltham, MA, USA) and qPCR reagent (TB Green^®^ Premix Ex Taq™ II reagent, RR820Q, Takara, Shanghai, China), with GAPDH used as the internal control. Three independent replicates were adopted, and the relative expression of the genes was calculated using the 2^−ΔΔCt^ method.

### 4.11. Data Analysis

Statistical analysis and graphing were performed using Excel 2010, SPSS 26.0, and GraphPad Prism 10.1.2. Multiple comparisons were conducted using one-way analysis of variance (ANOVA) followed by Duncan’s test. Different letters above the bars in the figures indicate significant differences, which were determined at *p* < 0.05.

## 5. Conclusions

This study analyzed the transcriptomic response of maize roots at different times following excessive Zn treatment and found that differentially expressed genes (DEGs) were enriched in the phenylpropanoid–lignin metabolic pathway. Subsequent analysis showed that the phenylpropanoid–lignin pathway responds during the early stages of excessive Zn treatment and functions continuously in Zn detoxification. Correspondingly, increased POD enzyme activity and lignin content further demonstrated the significant role of the phenylpropanoid–lignin metabolic pathway in the root’s response to Zn stress. These findings provide new insights into the mechanisms of adaptation to excessive Zn stress in maize.

## Data Availability

The datasets presented in this study can be found in online repositories. These datasets have been deposited in the Genome Sequence Archive of the National Genomics Data Center, Beijing Institute of Genomics, Chinese Academy of Sciences and China National Center for Bioinformation, under accession number GSA: CRA025463 (https://bigd.big.ac.cn/gsa/browse/CRA025463 (accessed on 9 May 2025)).

## Supplementary Materials

The following supporting information can be downloaded at: https://www.mdpi.com/article/10.3390/plants14111657/s1, Figure S1: Correlation analysis of RNA-seq data between samples. Samples are treated with 150 μM Zn for 0 h (R0), 6 h (R2), 12 h (R3), 24 h (R4), and 48 h (R5), with three biological replicates per group; Figure S2: Distribution of the gene expression; Figure S3: KEGG metabolic enrichment analysis of 89 common DEGs in ERS, MRS, and LRS; Figure S4: Expression of DEGs in maize roots related to Flavonoid metabolic pathway. (A) Metabolic diagram of Flavonoid metabolic pathway; (B) Expression changes of DEGs in Flavonoid metabolic pathway at different stages of Zn treatment; Figure S5: Expression changes of DEGs encoding potential Zn transporters and chelators in maize root; Table S1: Statistics of transcriptome sequencing data; Table S2: KEGG enrichment analysis of all DEGs in response to Zn treatment; TableS3: GO enrichment analysis of cell wall-related pathways in response to Zn treatment; Table S4: Primers used in this study.
