# Supplementary material for: Transcriptomic Profiling Reveals the Involvement of the Phenylpropanoid–Lignin Pathway in the Response of Maize Roots to Zinc Stress
Source: Plants (Basel). 2025 May 29;14(11):1657. doi: 10.3390/plants14111657 (PMC12158060; doi:10.3390/plants14111657)
Supplement: Supplementary file 1 [file plants-14-01657-s001.zip › plants-3660600-supplementary.pdf]

Supplementary Figures and Tables

Title: Transcriptomic Profiling Reveals the involvement of Phenylpropanoid-lignin pathway in response to Zn stress of maize root

Authors: Ying Zhou, Tianyu Gu, Yan Gao, Jingtao Qu, Hongjian Zheng, Yuan Guan, Jiashi Peng

Supplementary Figures

Supplementary Figure S1. Correlation analysis of RNA-seq data between samples.

Supplementary Figure S2. Distribution of the gene expression.

Supplementary Figure S3. KEGG metabolic enrichment analysis of 89 common DEGs in ERS, MRS, and LRS.

Supplementary Figure S4. Expression of DEGs in maize roots related to Flavonoid metabolic pathway.

Supplementary Figure S5. Expression changes of DEGs encoding potential Zn transporters and chelators in maize root.

Supplementary Tables

Supplementary Table S1. Statistics of transcriptome sequencing data.

Supplementary Table S2. KEGG enrichment analysis of all DEGs in response to Zn treatment.

Supplementary TableS3. GO enrichment analysis of cell wall-related pathways in response to Zn treatment

Supplementary Table S4. Primers used in this study.

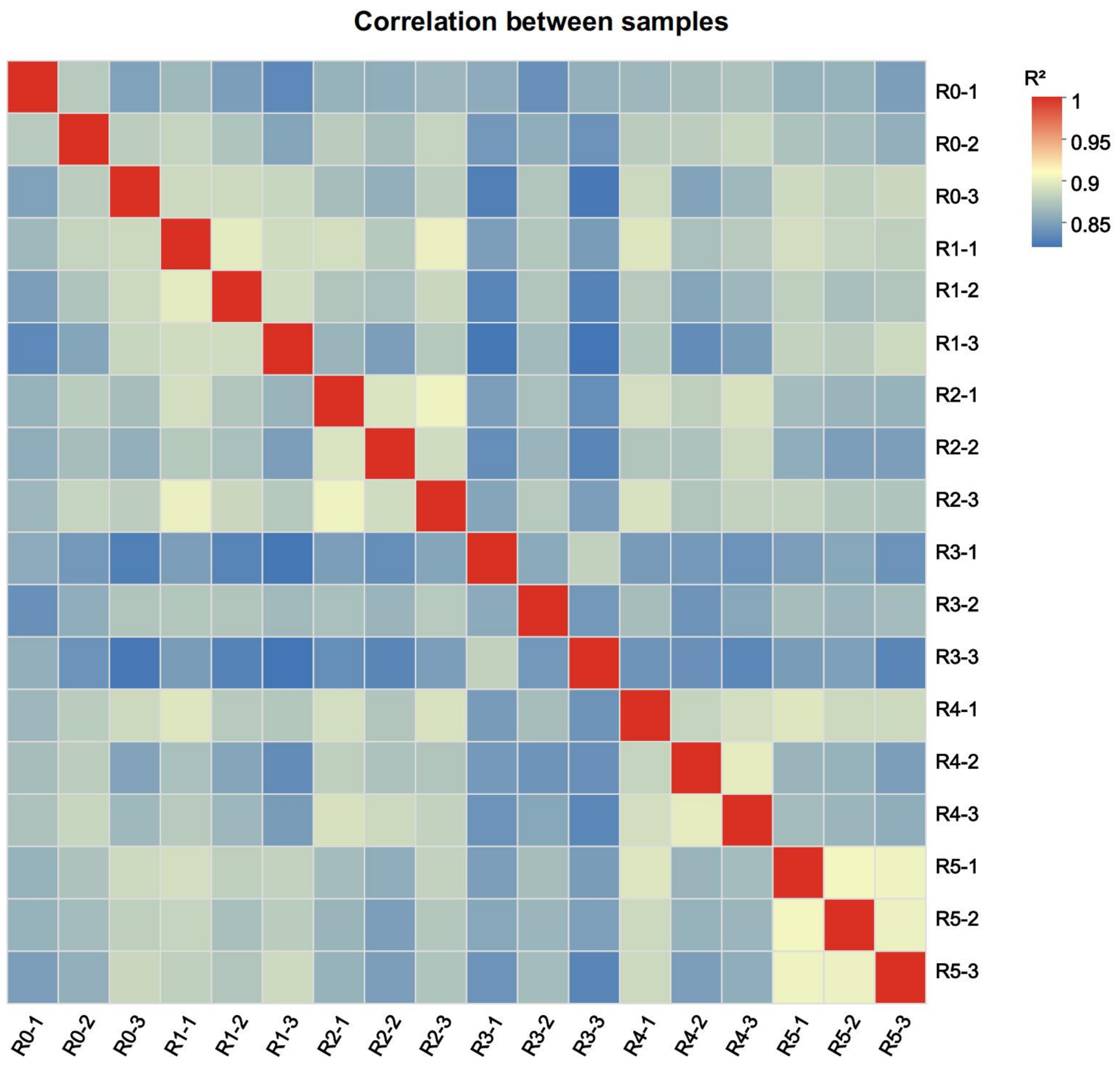

Supplementary Figure S1. Correlation analysis of RNA-seq data between samples. Samples are treated with 150 µM Zn for 0 h (R0), 6 h (R2), 12 h (R3), 24 h (R4), and 48 h (R5), with three biological replicates per group.

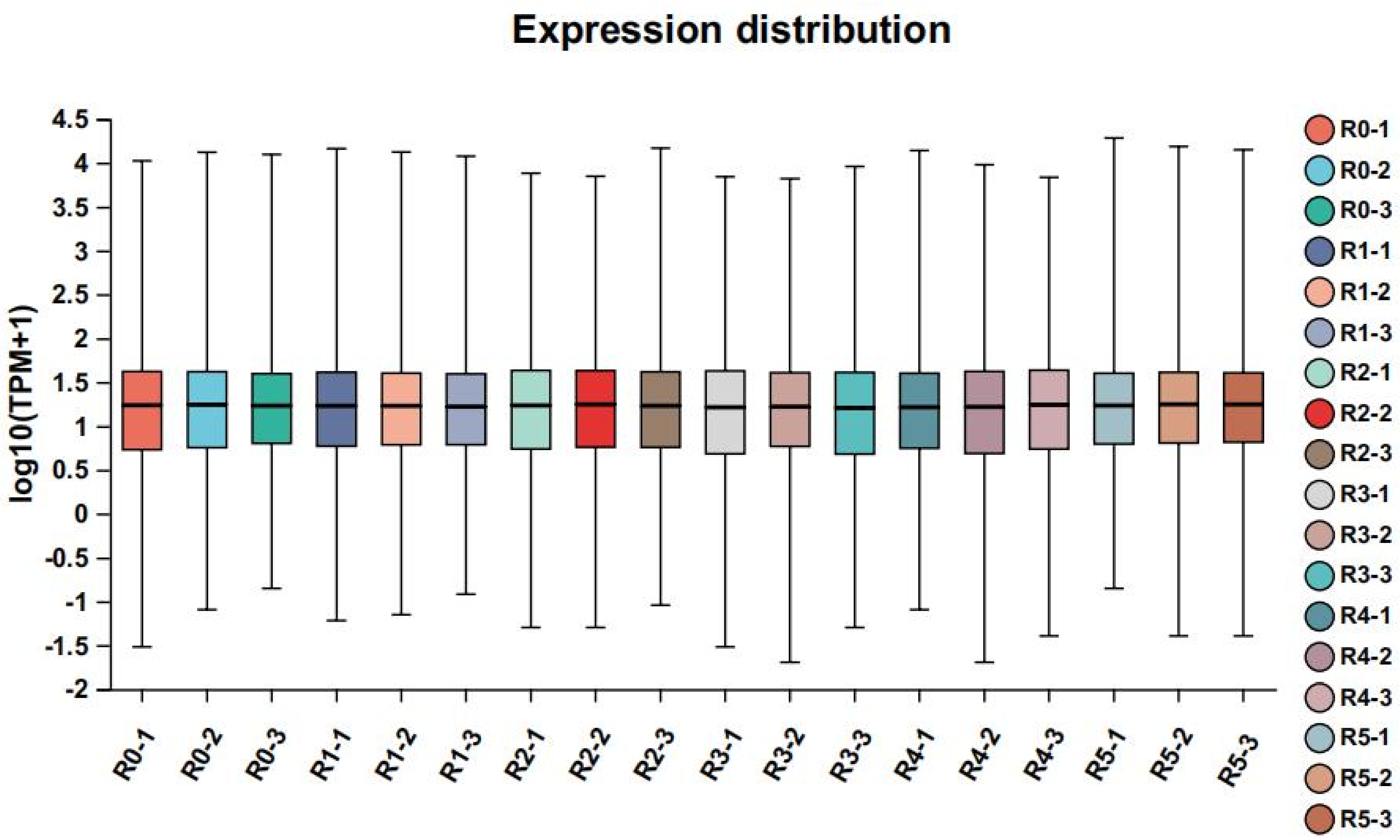

Supplementary Figure S2. Distribution of the gene expression.

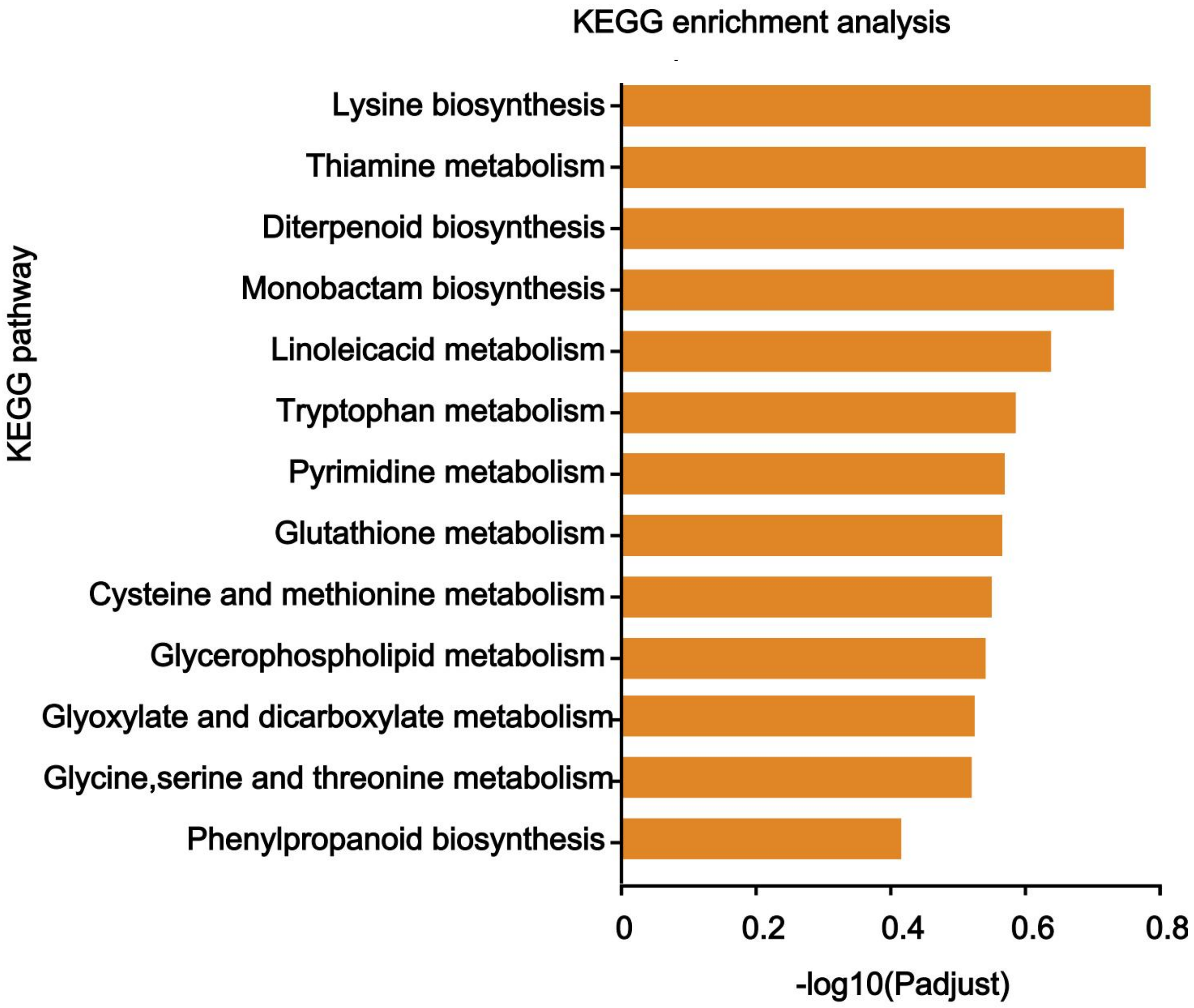

Supplementary Figure S3. KEGG metabolic enrichment analysis of 89 common DEGs in ERS, MRS, and LRS.

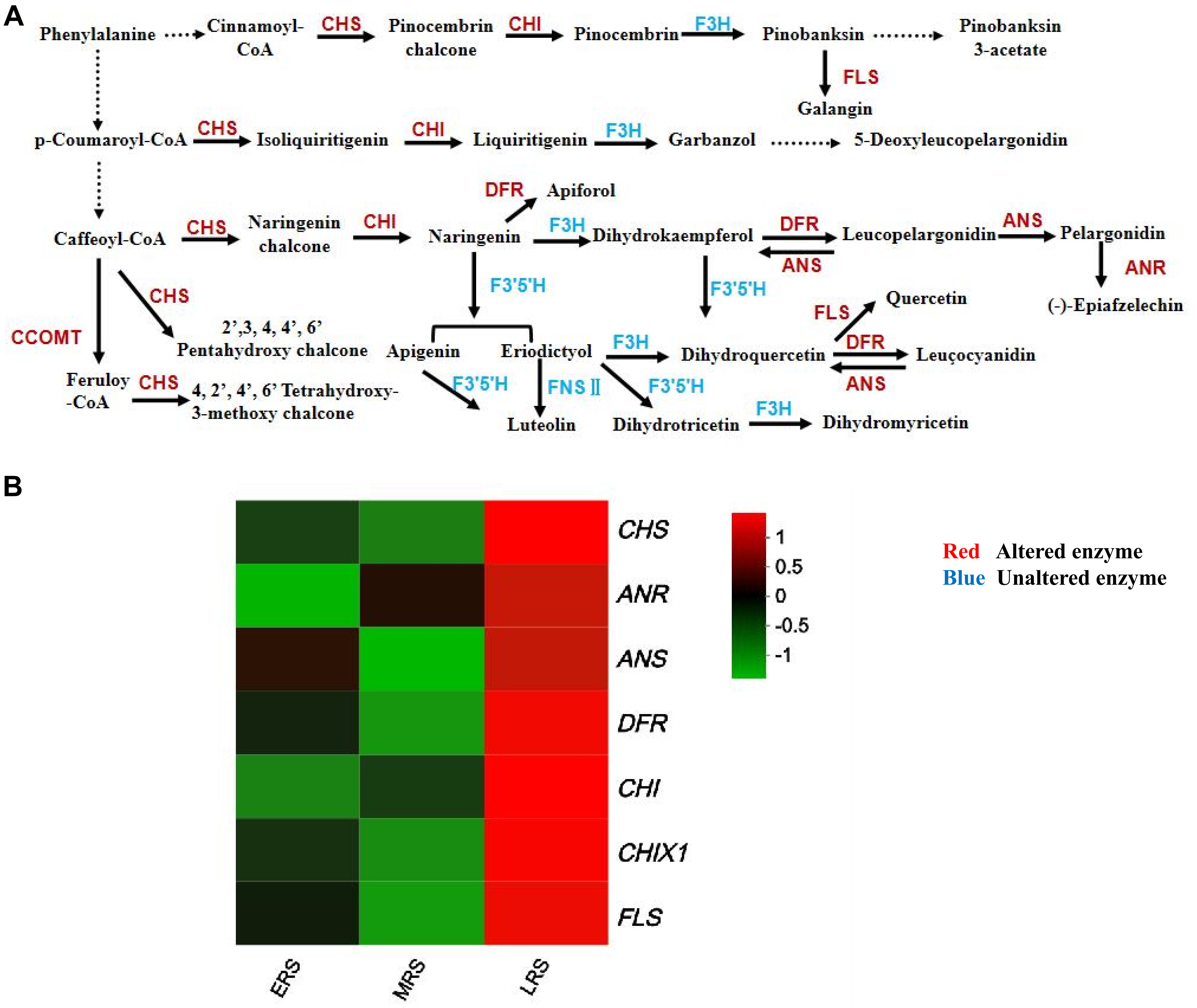

Supplementary Figure S4. Expression of DEGs in maize roots related to Flavonoid metabolic pathway. (A) Metabolic diagram of Flavonoid metabolic pathway; (B) Expression changes of DEGs in Flavonoid metabolic pathway at different stages of Zn treatment.

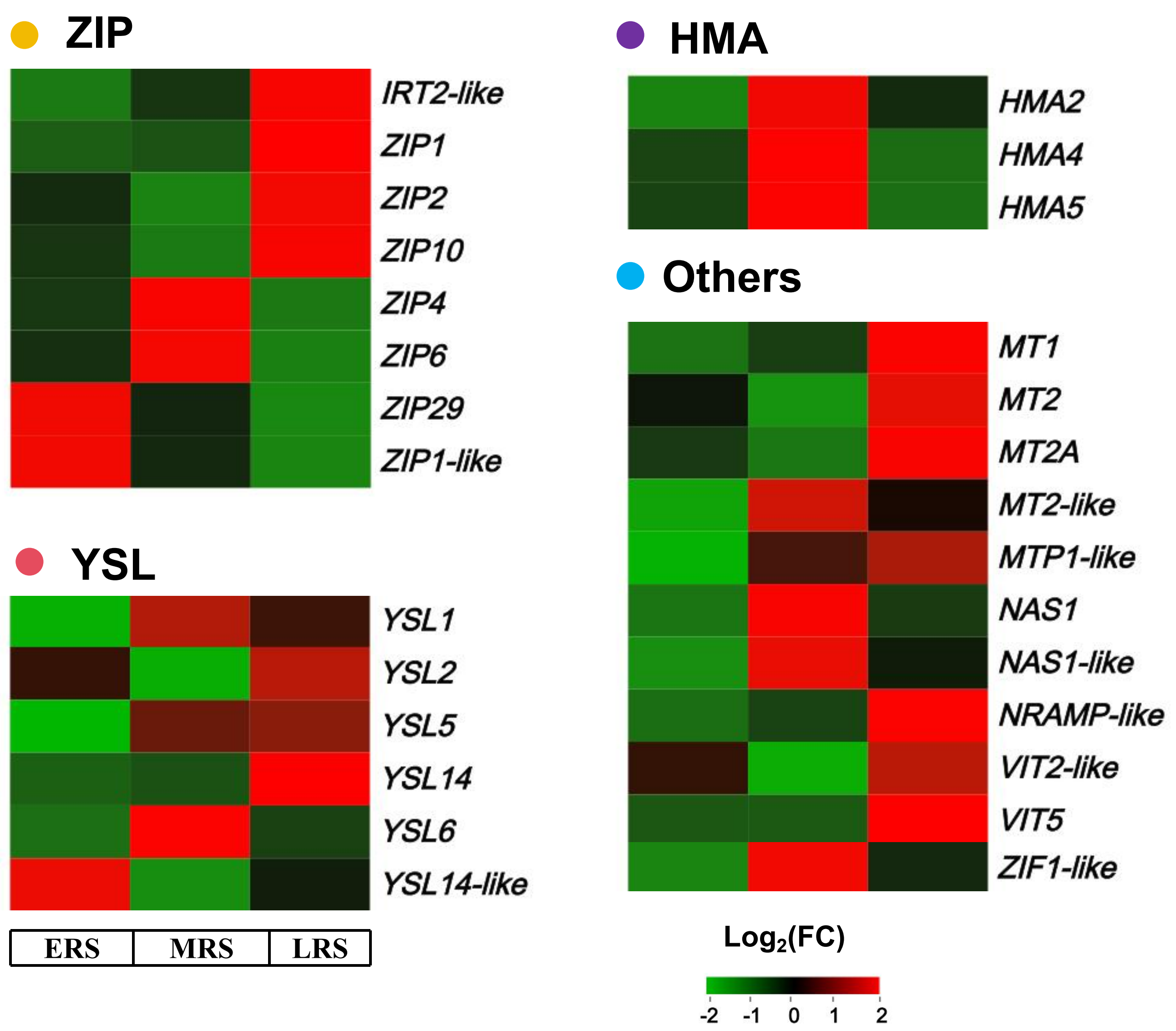

Supplementary Figure S5. Expression changes of DEGs encoding potential Zn transporters and chelators in maize root.

Supplementary Table S1. Statistics of transcriptome sequencing data.

Sample	Treatment time	Total base number	Clean read number	Total mapped	Q30 (%)	Q20 (%)	GC content (%)
R0-1	0h	49062044	48739112	46277624(94.95%)	94.59	98.31	48.43
R0-2	0h	50355032	50033662	48275308(96.49%)	94.37	98.24	48.7
R0-3	0h	53440998	53053074	51159364(96.43%)	94.42	98.22	49.94
R1-1	2h	57515502	57092008	54909480(96.18%)	94.11	98.13	49.42
R1-2	2h	45551894	45258188	43569763(96.27%)	94.46	98.26	49.55
R1-3	2h	50129646	49789132	47919442(96.24%)	94.25	98.16	50.57
R2-1	6h	43482858	43239278	41867897(96.83%)	94.68	98.36	48.38
R2-2	6h	46796670	46474100	44979369(96.78%)	94.45	98.27	48.02
R2-3	6h	52371316	51969856	50124291(96.45%)	94.42	98.24	49.04
R3-1	12h	56486976	56005018	53417000(95.38%)	94.21	98.15	49.38
R3-2	12h	47161328	46877296	45031458(96.06%)	94.19	98.16	49.01
R3-3	12h	52342592	51998284	50089429(96.33%)	94.17	98.14	49.63
R4-1	24h	55129948	54788764	52364347(95.57%)	94.5	98.28	49.25
R4-2	24h	44217484	43960406	42306131(96.24%)	94.24	98.2	48.3
R4-3	24h	54828590	54527386	52222017(95.77%)	94.46	98.28	47.72
R5-1	48h	61802702	61389662	58356128(95.06%)	94.46	98.24	50.04
R5-2	48h	55318182	54985524	52418432(95.33%)	94.42	98.23	50.06
R5-3	48h	59665150	59312130	56337254(94.98%)	94.38	98.22	50.32

This table only lists the pathways significantly altered by Zn treatment (P-adjust < 0.05)

Supplementary Table S2. KEGG enrichment analysis of all DEGs in response to Zn treatment.

Number	Pathway ID	Description	Ratio in study	Rich factor	*P*-adjust
123	map00940	Phenylpropanoid biosynthesis	123/ 2274	0.39	4.73E-30
59	map00480	Glutathione metabolism	59/ 2274	0.32	3.40E-10
29	map00592	alpha-Linolenic acid metabolism	29/ 2274	0.38	9.55E-7
27	map00945	Stilbenoid, diarylheptanoid and gingerol biosynthesis	27/ 2274	0.39	1.16E-6
13	map00591	Linoleic acid metabolism	13/ 2274	0.50	0.00013
60	map00500	Starch and sucrose metabolism	60/ 2274	0.23	0.00015
29	map04712	Circadian rhythm - plant	29/ 2274	0.29	0.00027
94	map04075	Plant hormone signal transduction	94/ 2274	0.19	0.00098
65	map04016	MAPK signaling pathway - plant	65/ 2274	0.20	0.0013
11	map00902	Monoterpenoid biosynthesis	11/ 2274	0.44	0.0014
9	map00402	Benzoxazinoid biosynthesis	9/ 2274	0.43	0.0067
21	map00130	Ubiquinone and other terpenoid-quinone biosynthesis	21/ 2274	0.27	0.0075
38	map00270	Cysteine and methionine metabolism	38/ 2274	0.26	0.0076
10	map00905	Brassinosteroid biosynthesis	10/ 2274	0.33	0.026
12	map00196	Photosynthesis - antenna proteins	12/ 2274	0.30	0.027
18	map00910	Nitrogen metabolism	18/ 2274	0.24	0.037
32	map00630	Glyoxylate and dicarboxylate metabolism	32/ 2274	0.20	0.049
25	map00250	Alanine, aspartate and glutamate metabolism	25/ 2274	0.21	0.043
15	map00906	Carotenoid biosynthesis	15/ 2274	0.25	0.043
13	map00904	Diterpenoid biosynthesis	13/ 2274	0.27	0.043
38	map00195	Photosynthesis	38/ 2274	0.19	0.048

This table only lists the pathways significantly altered by Zn treatment (*P*-adjust < 0.05)

Supplementary TableS3. GO enrichment analysis of cell wall-related pathways in response to Zn treatment

Number	GO ID	Description	Ratio_in_study	Rich factor	P-adjust
142	GO:0005618	cell wall	142/ 5187	0.29	6.05E-17
96	GO:0004601	peroxidase activity	96/ 5187	0.32	1.07E-14
58	GO:0009698	phenylpropanoid metabolic process	58/ 5187	0.37	1.42E-11
56	GO:0009505	plant-type cell wall	56/ 5187	0.31	3.00E-08
29	GO:0009808	lignin metabolic process	29/ 5187	0.45	5.98E-08
152	GO:0005976	polysaccharide metabolic process	152/ 5187	0.21	2.02E-06
65	GO:0071669	plant-type cell wall organization or biogenesis	65/ 5187	0.26	5.01E-06
28	GO:0009699	phenylpropanoid biosynthetic process	28/ 5187	0.38	6.07E-06
18	GO:0016762	xyloglucan:xyloglucosyl transferase activity	18/ 5187	0.49	9.74E-06
84	GO:0035251	UDP-glucosyltransferase activity	84/ 5187	0.23	1.12E-05
67	GO:0044036	cell wall macromolecule metabolic process	67/ 5187	0.24	8.52E-05
56	GO:0010410	hemicellulose metabolic process	56/ 5187	0.25	9.14E-05
66	GO:0000272	polysaccharide catabolic process	66/ 5187	0.24	1.02E-04
65	GO:0042546	cell wall biogenesis	65/ 5187	0.24	1.50E-04
57	GO:0010383	cell wall polysaccharide metabolic process	57/ 5187	0.24	2.53E-04
24	GO:0009664	plant-type cell wall organization	24/ 5187	0.33	4.72E-04
122	GO:0071554	cell wall organization or biogenesis	122/ 5187	0.19	9.12E-04
13	GO:0009809	lignin biosynthetic process	13/ 5187	0.43	1.33E-03
12	GO:0046274	lignin catabolic process	12/ 5187	0.40	4.94E-03
94	GO:0071555	cell wall organization	94/ 5187	0.19	5.16E-03
29	GO:0010411	xyloglucan metabolic process	29/ 5187	0.26	5.80E-03
41	GO:0009832	plant-type cell wall biogenesis	41/ 5187	0.23	8.57E-03
18	GO:0016998	cell wall macromolecule catabolic process	18/ 5187	0.31	9.86E-03
11	GO:0044347	cell wall polysaccharide catabolic process	11/ 5187	0.35	2.10E-02
11	GO:0045493	xylan catabolic process	11/ 5187	0.35	2.10E-02
26	GO:0009834	plant-type secondary cell wall biogenesis	26/ 5187	0.25	2.90E-02

Supplementary Table S4. Primers used in this study.

Gene names	Primer sequences (5′→3′)
GAPDH-f	ctggtttctaccgacttccttg
GAPDH-r	cggcatacacaagcagcaac
Peroxidase 2-f	ctcctcctcctcctcctccaacta
Peroxidase 2-r	gttcctcacggcttgcttgatga
Peroxidase3-f	ctcataacagcgttcggcaacaag
Peroxidase3-r	ttggtgtcgttgtagatgtggtcc
Peroxidase50-f	gcctccgacaagcaggacctgatga
Peroxidase50-r	acgtctcccggtacttcttcgccac
Peroxidase66-f	tgccttattagcctgtcgtcgttgg
Peroxidase66-r	agttcacgccgctcctgatggt
BGL-f	ctcatcaccgacacgctcaaga
BGL-r	tgatggagtggtagtagtgctggt
UGT-f	cgcttcttgggctccatcatctg
UGT-r	tcctcggcagcagcttgttct
